# Effect of an early neurocognitive rehabilitation on autonomic nervous system in critically ill patients

**DOI:** 10.1186/2197-425X-3-S1-A989

**Published:** 2015-10-01

**Authors:** M Turon, D Hernando, S Fernandez-Gonzalo, R Bailón, G Gomà, J Lázaro, J Montanyà, E Gil, M Martinez-Perez, C de Haro, J López-Aguilar, A Martinez-Rubio, M Jodar, P Laguna, L Blanch

**Affiliations:** Institut Universitari, Universitat Autònoma de Barcelona, Research Department, Fundació Parc Taulí, Sabadell, Spain; BSICOS Group, 13 A, University of Zaragoza & CIBER-BBN, Zaragoza, Spain; Hospital Universitari, Universitat Autònoma de Barcelona, Critical Care Department, Parc Taulí Sabadell, Sabadell, Spain; Centro de Investigación Biomédica en Red de Enfermedades Respiratorias, Instituto de Salud Carlos III, Sabadell, Spain; Hospital Universitari, Universitat Autònoma de Barcelona, Cardiology Depatment, Parc Taulí Sabadell, Sabadell, Spain; Hospital Universitari, Universitat Autònoma de Barcelona, Neurology Department, Parc Taulí Sabadell, Sabadell, Spain

## Introduction

Recent clinical and electrophysiological studies reveal a high incidence of autonomic nervous system (ANS) dysfunction in patients treated in ICU [1]. ANS disturbances may produce diverse and unexpected consequences. For instance, critically ill patients are at risk of neurocognitive impairments that may persist after hospital discharge. Among various pathophysiological mechanisms proposed, ANS dysfunction leading cholinergic deficiency seems one of the most viable to explain the development of long-term sequelae. Heart rate variability (HRV) has been related to the activity of the prefrontal cortex [2] hence, prefrontal activation could help to strengthen the autonomic nervous system integrity. We are interested in assessing the improvement of the ANS dysfunction through neural circuits' activation. Thus, we propose a novel therapy that could allow the reinforcing of ANS through an early neurocognitive intervention targeted to improve prefrontal activation.

## Objectives

The aim of this study was to explore if the integrity of the ANS, via cardiac vagal tone, measured by the HRV can be modified after early neurocognitive rehabilitation in ICU patients.

## Methods

A total of 17 critically ill patients received a 20-minute Early Neurocognitive Rehabilitation (ENR) session in their own bed in the ICU. HRV was derived from the recorded ECG signal during pre-session, session and post-session. Power in the specific frequency bands related to sympathetic and parasympathetic systems was computed (PLF and PHF for low and high frequency bands, respectively). PLF was computed within the classic band, while PHF was computed within a band centered at respiratory rate. Changes in the HRV parameters from pre-session to session, and from pre-session to post-session were studied using Wilcoxon signed-rank test.

## Results

Clinical data of the sample are summarized in table 1. Comparing with baseline values, 9 patients (53%) showed a decreased PLF in post-session, while 8 patients (47%) presented a higher PLF (p = .759). In 12 patients (71%), PHF increased after the ENR session, suggesting an increase of parasympathetic activity (p = .836).

**Table 1:** Descriptives.

Age (M, SD)		64,31	10.47
	*Male*	12	70,59
	*Female*	5	29, 41
Diagnosis (N, %)			
	*Intestinal perforation*	3	17, 65
	*Peritonitis*	3	17, 65
	*Septic shock*	2	11, 76
	*Politrauma*	2	11, 76
	*Pneumonia*	2	11, 76
	*Hemorrhagic shock*	1	5, 88
	*Toxic intake*	1	5, 88
	*Pancreatitis*	1	5, 88
	*Esophageal perforation*	1	5, 88
	*Acute respiratory failure*	1	5, 88
APACHE-II (M, SD)		24, 31	9, 53
SOFA (M, SD)		9, 5	4, 53
RASS (M, SD)		1, 13	2, 26
Length of ICU stay, days (M, SD)		24, 94	30, 17
Duration of intubation, days (M, SD)		18, 69	31, 27
Duration of sedation, days (M, SD)		7, 69	8, 55
Duration of Delirium during ICU (M, SD)		19, 85	34, 09
Septic Shock (N, %)		9	52, 94
Cardiac arrest (N, %)		1	5, 88
APACHE-II: Acute Physiology and Chronic Health Evaluation II; SOFA: Sequential Organ Failure Assessment: RASS: Richmond Agitation-Sedation Scale; MBS: Modified Borg Scale; M. Mean; SD: Standard Deviation.			

## Conclusions

Diagnosis, severity of illness or medication could explain the differential effect in the evolution of the HRV parameters among different patients. Despite differences, an early neurocognitive rehabilitation seems to increase parasympathetic activity after the session in the majority of the patients. Clinical characteristics of the critical ill patients should be further studied to determinate which patients could be the best candidates for early neurocognitive interventions
